# Impact of intravenous vitamin C as a monotherapy on mortality risk in critically ill patients: A meta-analysis of randomized controlled trials with trial sequential analysis

**DOI:** 10.3389/fnut.2023.1094757

**Published:** 2023-03-24

**Authors:** Kuo-Chuan Hung, Min-Hsiang Chuang, Jen-Yin Chen, Chih-Wei Hsu, Chong-Chi Chiu, Ying-Jen Chang, Chia-Wei Lee, I-Wen Chen, Cheuk-Kwan Sun

**Affiliations:** ^1^School of Medicine, College of Medicine, National Sun Yat-sen University, Kaohsiung City, Taiwan; ^2^Department of Anesthesiology, Chi Mei Medical Center, Tainan City, Taiwan; ^3^Department of Internal Medicine, Chi Mei Medical Center, Tainan City, Taiwan; ^4^Department of Psychiatry, Kaohsiung Chang Gung Memorial Hospital, Chang Gung University College of Medicine, Kaohsiung City, Taiwan; ^5^Department of General Surgery, E-Da Cancer Hospital, I-Shou University, Kaohsiung City, Taiwan; ^6^School of Medicine, College of Medicine, I-Shou University, Kaohsiung City, Taiwan; ^7^Department of Medical Education and Research, E-Da Cancer Hospital, I-Shou University, Kaohsiung City, Taiwan; ^8^Department of Neurology, Chi Mei Medical Center, Tainan City, Taiwan; ^9^Department of Anesthesiology, Chi Mei Medical Center, Liouying, Tainan City, Taiwan; ^10^Department of Emergency Medicine, E-Da Hospital, I-Shou University, Kaohsiung City, Taiwan

**Keywords:** vitamin C, sepsis, septic shock, critically illness, mortality

## Abstract

**Background:**

This meta-analysis aimed at investigating the pooled evidence regarding the effects of intravenous vitamin C (IVVC) on mortality rate in critically ill patients.

**Methods:**

Databases including Medline, Embase, and Cochrane Library were searched from inception to October, 2022 to identify RCTs. The primary outcome was the risk of overall mortality. Subgroup analyses were performed based on IVVC dosage (i.e., cut-off value: 100 mg/kg/day or 10000 mg/day). Trial sequential analysis (TSA) was used to examine the robustness of evidence.

**Results:**

A total of 12 trials including 1,712 patients were analyzed. Although meta-analysis demonstrated a lower risk of mortality in patients with IVVC treatment compared to those without [risk ratio (RR): 0.76, 95% CI: 0.6 to 0.97, *p* = 0.02, *I*^2^ = 36%, 1,711 patients), TSA suggested the need for more studies for verification. Moreover, subgroup analyses revealed a reduced mortality risk associated with a low IVVC dosage (RR = 0.72, *p* = 0.03, 546 patients), while no beneficial effect was noted with high IVVC dosage (RR = 0.74, *p* = 0.13, *I*^2^ = 60%, 1,165 patients). The durations of vasopressor [mean difference (MD): −37.75 h, 404 patients) and mechanical ventilation (MD: −47.29 h, 388 patients) use were shorter in the IVVC group than those in the controls, while there was no significant difference in other prognostic outcomes (e.g., length of stay in intensive care unit/hospital) between the two groups.

**Conclusion:**

Although intravenous vitamin C as a monotherapy reduced pooled mortality, durations of vasopressor use and mechanical ventilation, further research is required to support our findings and to identify the optimal dosage of vitamin C in the critical care setting.

**Systematic review registration:**

https://www.crd.york.ac.uk/prospero/, identifier CRD42022371090.

## 1. Introduction

Vitamin C, also known as ascorbic acid or ascorbate, has been reported to play a beneficial role in critical illnesses including sepsis as well as life-threatening cardiovascular diseases including coronary heart disease and stroke (1–5). Not only have previous studies demonstrated its ability to restore vasopressor sensitivity and preserve vascular endothelial integrity (2, 6–8), but vitamin C is also known to reinforce both innate and adaptive immunity (9). Therefore, the potential therapeutic benefits of vitamin C supplementation have been investigated in patients subjected to a variety of critical care settings including those with sepsis, those undergoing cardiac surgery, and those with COVID-19 infection (10–13). Nevertheless, evidence regarding the efficacy of vitamin C against risk of mortality remains to be clarified. Several meta-analyses reported no beneficial effect of vitamin C on the risk of mortality in cardiac surgical patients and those with COVID-19 infection (14, 15). For patients with sepsis/septic shock or critical illnesses, the association between mortality and the use of vitamin C was also controversial (16–22). Such conflicting findings may be attributed to the variations among individual studies including study design (i.e., prospective or retrospective), the choice of study population (e.g., critical vs. non-critical), the use of vitamin C as monotherapy or part of a combined regimen, as well as the selected dosage and route of administration.

Focusing on the critically ill population receiving intravenous vitamin C (IVVC) as a monotherapy, previous meta-analyses suggested that the use of IVVC may reduce the risk of mortality without a positive impact on the length of stay (LOS) in hospital or intensive care unit (ICU) (16, 17). However, the inclusion of a limited number of patients for analysis in the two meta-analyses [i.e., 467 patients (16) and 755 patients (17)] may impair the robustness of evidence. In contrast, a recent large-scale randomized controlled trial (RCTs) involving 872 patients with sepsis even reported a slight increase in the risk of mortality/persistent organ dysfunction at 28 days in those with IVVC monotherapy compared to those without (23).

In an attempt to provide clinical guidance based on updated information from RCTs, this meta-analysis aimed at evaluating the impact of IVVC as a monotherapy on the risk of mortality in critically ill adult patients and examining the robustness of evidence through performing trial sequential analyses (TSA).

## 2. Methods

### 2.1. Protocol and registration

Preferred Reporting Items for Systematic Reviews and Meta-Analyses have been followed in this meta-analysis, and the protocol has been registered with the International Prospective Register of Systematic Reviews (CRD42022371090).

### 2.2. Data sources and search strategy

We searched the databases of MEDLINE, EMBASE, and Cochrane Central Register of controlled trials to retrieve RCTs that fit our eligibility criteria from inception to October, 2022 without language restriction. As part of this analysis, only RCTs examining IVVC and prognostic outcomes in critically ill adult patients were included. The search strategies using subject headings and keywords are available in Supplementary Table 1. The reference lists of the retrieved articles and meta-analyses were also manually searched to identify potentially eligible articles that may have been overlooked during the initial digital search. The authors of articles with missing data of interest were emailed up to three times in an attempt to acquire the information.

### 2.3. Inclusion and exclusion criteria

Randomized controlled trials that met the following screening criteria were considered eligible for inclusion in the current study: (a) Population: Adults (age ≥ 18 years) who were critically ill [defined as those requiring ICU admission or those with a high rate of mortality (i.e., >5%) in the control arm as previously described (16)], (b) Intervention: IVVC as a monotherapy irrespective of the duration or the dosage of the treatment (IVVC group), (c) Comparison: Use of placebo or standard care (i.e., control group), (d) Outcomes: Availability of mortality risk. Other prognostic outcomes (e.g., duration of vasopressor use) were also analyzed if available.

Exclusion criteria were: (1) IVVC as combined therapy with other agents (e.g., hydrocortisone or thiamine); (2) studies without control group; (3) those adopting oral vitamin C supplementation as an intervention group; (4) those published without peer-review or as abstracts only; (5) those with research designs other than RCT (e.g., case reports or reviews); and (6) those focusing on patients undergoing cardiac surgery.

### 2.4. Study selection and data extraction

The records obtained from database search were screened as follows. After independent assessment of the titles and abstracts of the articles by two independent reviewers, the same reviewers read the full texts to determine their eligibility. Other reviewers then independently extracted relevant data including country, first author’s name, publication year, participant-related information (e.g., age, gender, and sample size), information regarding pharmacological therapies (e.g., dosage and treatment duration), and outcome-associated details (e.g., mortality rate, hospital LOS). In the event that missing information was needed, we emailed the corresponding authors in an attempt to get access to the data.

### 2.5. Clinical outcomes

The primary outcome was the risk of overall mortality. Secondary outcomes included durations of vasopressor use and mechanical ventilation time, risk of renal replacement therapy, ICU/hospital LOS as well as changes in circulating C-reactive protein (CRP) concentration and sequential organ failure assessment (SOFA) score. Subgroup analyses were performed based on the indications of IVVC treatment and dosage of IVVC (i.e., low vs. high) with cut-off value being set at 100 mg/kg/day or 10000 mg/day as previously reported (17).

### 2.6. Assessment of methodologic quality

Each trial was assessed for bias using the criteria outlined in the Cochrane Handbook for Systematic Reviews of Interventions by two authors (24). Disagreements were resolved through discussion. The potential risks of bias of individual studies were rated as “low,” “high,” or “unclear.”

### 2.7. Data synthesis

We used RevMan 5.4 (Cochrane IMS, Oxford, United Kingdom) with a random effects model as the basis for all analyses. The pooled risk ratios for dichotomous data are presented as risk ratios (RR), while continuous variables are presented as weighted mean differences (MD). All estimates are provided with 95% confidence intervals (CI). A funnel plot was generated to assess the possibility of publication bias if an outcome was reported in ten or more studies. Egger regression test was used in case of funnel plot asymmetry if required. The potential influence of the findings of an individual trial on the overall results was evaluated with sensitivity analysis using a “leave-one-out” approach. For all analysis, *p*-value of less than 0.05 was considered statistically significant.

To assess the strength of the results and to guard against statistical errors of type I and type II, we performed trial sequential analysis (TSA) as previously described (25, 26). We calculated the diversity-adjusted information size using 80% power while maintaining an overall two-sided type I error of 5%. Using this method, we could determine whether the conclusion was sufficient or if further studies are required to detect a predefined 20% reduction in the risk of overall mortality. The analysis was conducted using TSA software, Copenhagen Trial Unit version 0.9.5.10 Beta.

## 3. Results

### 3.1. Characteristics of trials and risk of bias

**FIGURE 1:**
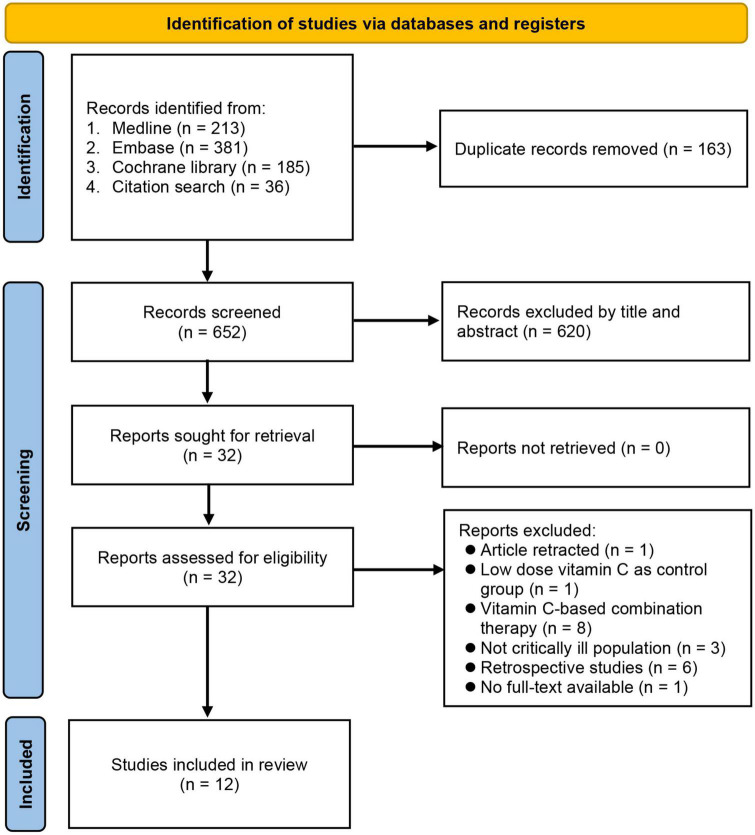
PRISMA flow diagram of study selection for the current meta-analysis.

Through electronic (*n* = 779) and manual (*n* = 36) searches, 815 records were identified. Following deletion of duplicates (*n* = 163) and reports deemed unsuitable after title and abstract screening (*n* = 620), 32 articles were considered eligible for full-text reading that further excluded 20 studies. Finally, 12 studies with 1,712 critically ill patients published between 2014 and 2022 were included in the current meta-analysis (23, 27–37) (Figure 1). Among the included studies, one RCT had two intervention arms (i.e., 50 or 200 mg/kg/day); therefore, the results of that study were analyzed separately [i.e., Fowler (28) and Hill et al. (15)].

The characteristics of the eligible studies are shown in Table 1. The study population included 1,712 patients with sepsis/septic shock (eight trials) (23, 27–29, 33–36), severe COVID-19 (three trials) (30, 31, 37), and severe pneumonia (one trial) (32). The number of patients in the included studies ranged from 28 to 863. Of all patients, the male proportion was 50–80% and the average or median age was 30–92 years. Nine studies provided detail on the baseline SOFA score (23, 28, 29, 32–37), while related information was not available in two trials (30, 31) and in another study that assessed the risk of mortality using the Physiological and Operative Severity Score for the enumeration of Mortality and morbidity (POSSUM) score (27). High-dose IVVC (i.e., ≥100 mg/kg/day or 10000 mg/day) was used in six studies (23, 28, 29, 34, 36, 37), while low-dose IVVC was applied in seven trials (27, 28, 30–33, 35). The overall mortality rates were 29% (range: 9–60%) and 32.6% (range: 10–64%) in the IVVC and control groups, respectively.

**TABLE 1:** Characteristics of studies (*n* = 12).

References	Population	Age (years)^#^	Male (%)^#^	Baseline SOFA score^#^	Vitamin C group	Placebo group	Total *N*	Mortality (%)	Country
Ferrón-Celma et al. (27)	Patients with sepsis	68 vs. 65	50 vs. 60	50 vs. 50†	450 mg/day for 6 days	5% dextrose	20	60 vs. 40	Spain
Fowler et al. (28)	Patients with severe sepsis	30–70 49–92 54–68¶	63 50 50¶	10 11 13¶	50 or 200 mg/kg/day for 4 days	5% dextrose	24	44 vs. 63	USA
Fowler et al. (29)‡	Patients with sepsis and ARDS	54 vs. 57	54 vs. 54	9.8 vs. 10.3	200 mg/kg/day for 4 days	5% dextrose	167	30 vs. 46	USA
JamaliMoghadamSiahkali et al. (30)	Patients with severe COVID-19	58 vs. 61	50 vs. 50	NA	6 g/day for 5 days	Standard therapy§	60	10 vs. 10	Iran
Kumari et al. (31)	Patients with severe COVID-19	52 vs. 53	56.90%	NA	50 mg/kg/day and standard care	Standard therapy	150	9 vs. 15	Pakistan
Lamontagne et al. (23)	Patients with sepsis	65 vs. 65	65 vs. 60	10.2 vs. 10.1	200 mg/kg/day for 4 days	5% dextrose	863	35 vs. 32	Canada
Mahmoodpoor et al. (32)	Patients with severe pneumonia	57 vs. 58	57 vs. 58	12.5 vs. 10.7	60 mg/kg/day for 4 days	Normal saline	80	15 vs. 28	Iran
Nabil Habib and Ahmed (33)	Patients with septic shock	43 vs. 42	56 vs. 60	10.2 vs. 11.4	6 g/day until ICU discharge	Conventional sepsis treatment	100	24 vs. 36	Egypt
Rosengrave et al. (34)	Patients with septic shock	69 vs. 66	80 vs. 55	8.5 vs. 9	100 mg/kg/day for 4 days	5% dextrose	40	30 vs. 35	New Zealand
Wacker et al. (35)‡	Patients with septic shock	69 vs. 73	50 vs. 52	10 vs. 9	1000 mg bolus followed by 250 mg/h for 4 days	Normal saline	124	27 vs. 41	USA
Zabet et al. (36)	Patients with septic shock	64 vs. 64	71 vs. 79	11.8 vs. 12.4	100 mg/kg/day for 3 days	5% dextrose	28	14 vs. 64	Iran
Zhang et al. (37)‡	Patients with severe COVID-19	66 vs. 67	56 vs. 76	14 vs. 13	24 g/day for 7 days	Bacteriostatic water	56	22 vs. 34	China

‡Multicenter trial; ¶placebo group; †Physiological and Operative Severity Score for the enumeration of Mortality and morbidity (POSSUM); §lopinavir/ritonavir/hydroxychloroquine; ICU, intensive care unit; ^#^presented as vitamin C vs. control groups.

**FIGURE 2:**
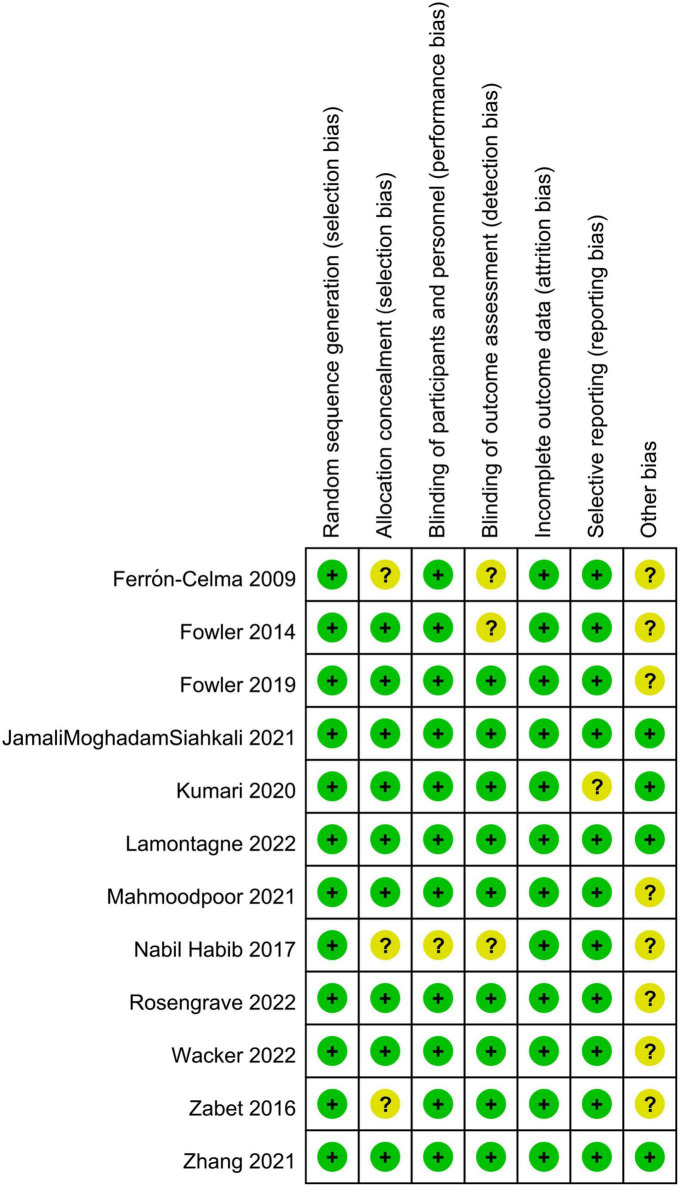
Risks of bias of the included randomized controlled trials.

No high-risk study was noted in any of the seven domains of bias assessment. All studies were deemed to have a low risk of selection bias because of their providing details about the methodology of randomization, while unclear risks of allocation concealment, performance bias, detection bias, reporting bias, other bias were considered in three, one, three, one, and eight trials, respectively (Figure 2).

### 3.2. Outcomes

#### 3.2.1. Primary outcome: Risk of mortality

**FIGURE 3:**
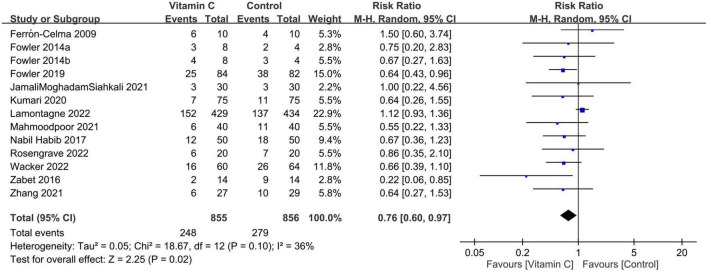
Forest plot comparing risk of mortality between intravenous vitamin C and control groups. M-H, Mantel-Haenszel; CI, confidence interval.

**FIGURE 4:**
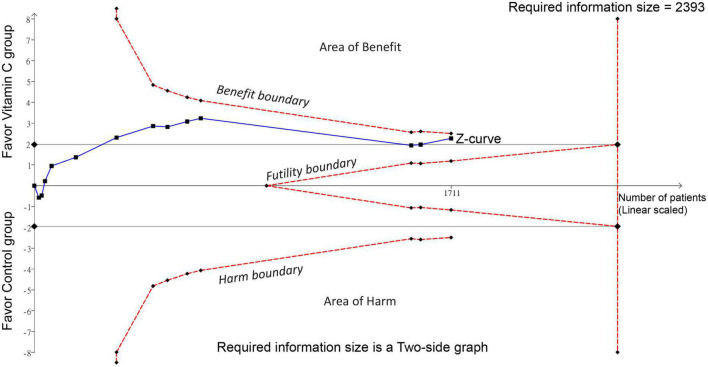
Trial sequential analysis (TSA) showing no crossing between the cumulative Z curve and the trial sequential monitoring boundary or the required information size, indicating insufficient and inconclusive evidence supporting the association of intravenous vitamin C supplementation with a decreased risk of mortality.

Forest plot demonstrated a lower risk of mortality in patients receiving IVVC as a monotherapy compared to those in the control group (RR: 0.76, 95% CI: 0.6 to 0.97, *p* = 0.02, *I*^2^ = 36%, 1,711 patients) (Figure 3) (23, 27–37). Sensitivity analysis revealed an inconsistent finding when three studies were removed one at a time (29, 35, 36). There was a low risk of publication bias (Supplementary Figure 1). TSA showed that the current evidence regarding the impact of IVVC on mortality rate remains inconclusive (Figure 4).

**FIGURE 5:**
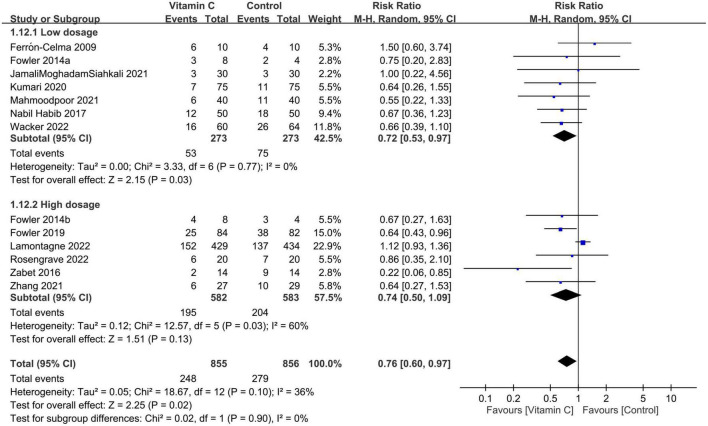
Subgroup analysis based on vitamin C dosage (cut-off value: 10000 mg/day or 100 mg/kg/day). M-H, Mantel-Haenszel; CI, confidence interval.

Despite the absence of subgroup difference based on dosage, our analysis demonstrated an association of a low dosage (i.e., <100 mg/kg/day or 10000 mg/day) of IVVC with a reduction in mortality risk (RR = 0.72, 95% CI: 0.53 to 0.97, *p* = 0.03, *I*^2^ = 0%, 546 patients), but without a positive impact for a high dosage (RR = 0.74 95% CI: 0.5 to 1.09, *p* = 0.13, *I*^2^ = 60%, 1,165 patients) (Figure 5). On the other hand, albeit statistically non-significant after being separated according to indications, the findings demonstrated a trend in favor of using vitamin C to reduce the risk of mortality in both subgroups based on treatment indications (i.e., sepsis/septic shock vs. COVID-19/pneumonia) (Supplementary Figure 2).

#### 3.2.2. Secondary outcome: Impact of vitamin C on vasopressor use, mechanical ventilation time, and risk of renal replacement therapy

**FIGURE 6:**
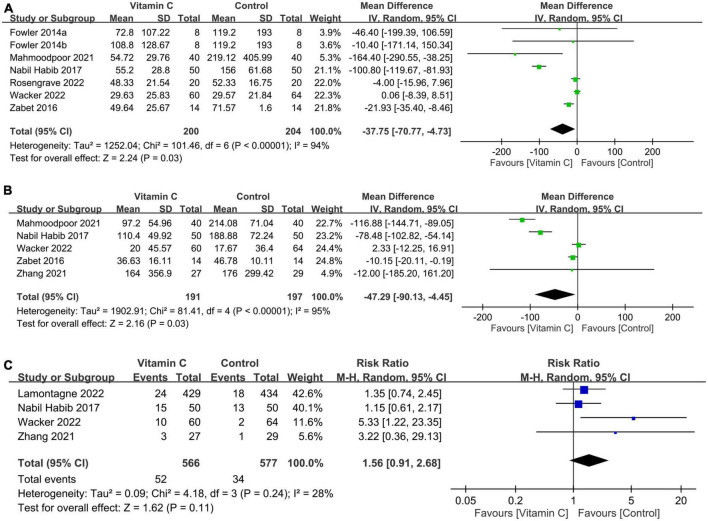
Forest plot comparing **(A)** duration of vasopressor use; **(B)** mechanical ventilation time; and **(C)** risk of renal replacement therapy between the intravenous vitamin C and control groups. CI, confidence interval; IV, inverse variance; M-H, Mantel-Haenszel.

The use of IVVC was associated with a shorter duration of vasopressor use than that in the controls (MD: −37.75 h, 95%: −70.77 to −4.73, *p* = 0.03, I^2^:94%, 404 patients) (Figure 6A) (28, 32–36). Nevertheless, the findings were inconsistent on sensitivity analysis. The duration of mechanical ventilation was also shorter in the IVVC group compared to that in the control group (MD: −47.29 h, 95% CI: −90.13 to −4.45, *p* = 0.03, *I*^2^ = 95%, 388 patients) (Figure 6B) (32, 33, 35–37) with inconsistent results on sensitivity analysis. The risk of renal replacement therapy was comparable between the two groups (RR = 1.56, 95% CI: 0.91 to 2.68, *p* = 0.11, *I*^2^ = 28%, 1,143 patients) (Figure 6C) (23, 33, 35, 37) with consistent findings on sensitivity analysis.

#### 3.2.3. Impact of IVVC on CRP level, SOFA score, and serum vitamin C level

**FIGURE 7:**
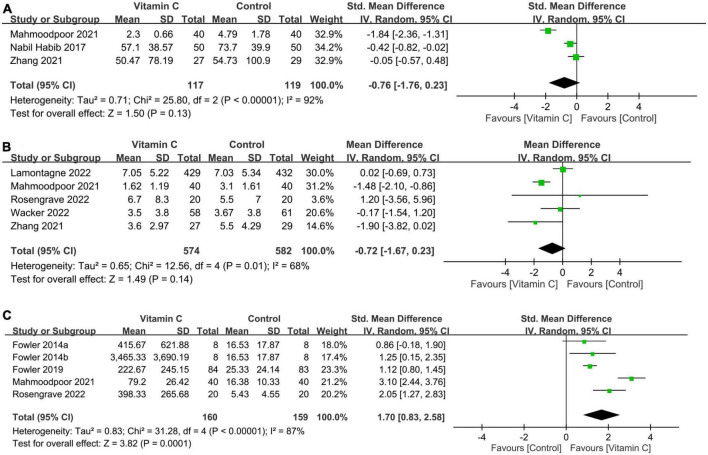
Forest plot comparing **(A)** circulating C-reactive protein concentration; **(B)** sequential organ failure assessment (SOFA) score; and **(C)** serum vitamin C level between intravenous vitamin C and control groups. CI, confidence interval; IV, inverse variance; Std, standardized mean difference.

There was no difference in CRP levels (MD: −0.76, 95% CI: −1.76 to 0.23, *p* = 0.13, *I*^2^ = 92%, 236 patients; sensitivity analysis: consistent) (Figure 7A) (32, 33, 37) and SOFA score (MD: −0.72, 95% CI: −1.67 to 0.23, *p* = 0.14, *I*^2^ = 68%, 1,156 patients) (Figure 7B) (23, 32, 34, 35, 37) between patients receiving IVVC and those subjected to control treatments. Sensitivity analysis revealed a lower SOFA score in the IVVC group compared to that in the control group when one study (23) was removed (MD: −1.12, 95% CI: −1.98 to −0.25, *p* = 0.01, *I*^2^ = 31%, 295 patients). The analysis of four studies providing information about serum vitamin C level demonstrated a higher serum vitamin C level in the IVVC group than that in the control group (SMD:1.7, 95% CI: 0.83 to 2.58, *p* = 0.0001, *I*^2^ = 87%, 319 patients; sensitivity analysis: consistent) (Figure 7C) (28, 29, 32, 34).

#### 3.2.4. Secondary outcomes: Hospital and ICU length of stay

**FIGURE 8:**
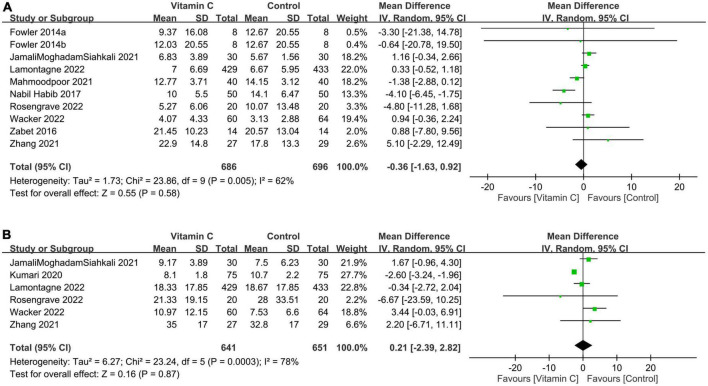
Forest plot comparing the **(A)** length of stay in the intensive care unit (ICU) and **(B)** length of hospital stay between the intravenous vitamin C and control groups. M-H, Mantel-Haenszel; CI, confidence interval.

There were no differences in ICU (MD: −0.36 days, 95% CI: −1.63 to 0.92, *p* = 0.58, *I*^2^ = 62%, 1,382 patients) (Figure 8A) (23, 28, 30, 32–37) and hospital (MD: 0.21 days, 95% CI: −2.39 to 2.82, *p* = 0.87, *I*^2^ = 78%, 1,292 patients) LOS (Figure 8B) (23, 30, 31, 34, 35, 37) between the IVVC and control groups. Sensitivity analysis showed consistent findings on these two outcomes. There was a low risk of publication bias on ICU LOS (Supplementary Figure 3).

## 4. Discussion

Our updated meta-analysis of 12 trials including 1,712 critically ill patients demonstrated an association between the use of IVVC and a reduced mortality rate. Nevertheless, TSA analysis suggested that further studies are required to provide conclusive evidence. Interestingly, we found that the correlation between mortality and IVVC may be influenced by the dosage of IVVC with a favorable outcome being noted with a low dosage of IVVC (i.e., <100 mg/kg/day or 10000 mg/kg/day). In addition, the durations of vasopressor and mechanical ventilation use were shorter in the IVVC group compared to those in the control group, while there was no significant difference in other prognostic outcomes (i.e., risk of renal replacement therapy, CRP level, SOFA score, hospital/ICU LOS) between the two groups.

The unexpected finding in a recent large-scale RCT showing a slightly higher risk of mortality and persistent organ dysfunction in patients with sepsis receiving IVVC (23) highlighted the need for updating and re-analyzing the pooled evidence. Compared with the control group, the current meta-analysis showed that IVVC monotherapy was associated with a lower mortality risk in critically ill patients. Although our TSA suggested the requirement for more trials to achieve a conclusive finding, our results demonstrated that the benefits of IVVC could outweigh the reported risks in the critically ill patients (23). Because previous meta-analyses reported no beneficial effect on the risk of mortality in septic patients subjected to vitamin C-based combination therapy and in those who underwent cardiac surgeries receiving intravenous/oral vitamin C monotherapies (15, 18), we only included the critically ill population (i.e., mortality rate in the control group was more than 5%) receiving IVVC as a monotherapy with the exclusion of cardiac surgical patients to minimize the potential bias. The I^2^ was only 36% in our primary outcome, suggesting a low heterogeneity in the current meta-analysis. Overall, our research provided additional information that could be used to guide clinical practice in the critical care setting.

Our subgroup analysis revealed that a low dosage of IVVC was associated with a reduced risk of mortality, while this benefit was not noted with a high dosage of IVVC. The current meta-analysis is the first to unveil this novel finding in the critically ill population. Although the underlying mechanism for this finding remains unclear, several previous observation studies and meta-analyses in different clinical settings have shown that a low-to-moderate dose of vitamin C may be more effective for decreasing the mortality rate than a high dose (38–40). For instance, a recent meta-analysis involving 19 studies on adults with COVID-infection reported that a low dosage (i.e., ≤1 g/day) of vitamin C could effectively decrease 1-month mortality, while no beneficial effect was observed with a large dosage of vitamin C (i.e., >1 g/day) (38). Another previous meta-analysis focusing on the use of IVVC monotherapy or vitamin C-based combination therapy found that vitamin C treatment for 3–4 days significantly improved the mortality rate in septic patients, while the risk of mortality in patients treated for 1–2 or >5 days was not reduced (40). Consistently, another large prospective cohort study (i.e., 28,945 participants) investigating the relationships between individual-level dietary intakes of vitamins C and all-cause mortality in the general population reported a lower all-cause mortality in individuals with moderate vitamin C intake (e.g., third and fourth quintiles) compared with those with the highest quintile of vitamin C intake (i.e., fifth quintiles) (39). Therefore, the lack of a dose-dependent effect between vitamin C supplement and the reduction in risk of mortality in those studies (38–40) indirectly supported the findings of the current meta-analysis. Regarding the impact of treatment indications, our findings of subgroup analysis suggest that while there may be some benefits of taking vitamin C, the effect was not statistically significant when each subgroup (i.e., sepsis/septic shock vs. COVID-19/pneumonia) was considered separately, probably due to a relatively small number of participants in each subgroup after being split.

Our results of shorter durations of vasopressor use in the IVVC group compared to those in the control group were consistent with those of previous meta-analyses (17, 19, 41). In contrast with the findings of other studies (19, 41, 42), we demonstrated no improvement in SOFA score associated with the monotherapeutic use of IVVC. Nevertheless, because of the high heterogeneity and inconsistent findings on sensitivity analysis of the three secondary outcomes, we considered our findings to be inconclusive. Further studies are required to address these issues. Another interesting finding of the current study was a non-significant trend toward a higher risk of renal replacement therapy linked to the use of IVVC compared with that in the control group based on data from four available trials that adopted relatively large IVVC dosages (i.e., 200 mg/kg/day, 6 g/day, 24 g/day) (23, 33, 35, 37) despite not fitting the criteria for a high dosage in this study. Despite the lack of robustness of evidence based on the small number of studies, our finding may be in line with that of a previous cohort study on 1,390 critically ill patients showing that IVVC with a dosage ≥1.5 g four times a day was associated with an increased risk of acute kidney and in-hospital mortality compared with those receiving no treatment or a single dose less than 1.5 g (43).

Consistent with the findings of several recent meta-analyses focusing on patients with sepsis/septic shock or critical illnesses that showed no difference in hospital/ICU stay with or without the use of vitamin C (16, 18, 19), the current meta-analysis focused on a similar population also demonstrated no difference in hospital/ICU stay with the use of IVVC as a monotherapy. In contrast, a previous meta-analysis on ICU patients reported a reduced ICU LOS with the use of intravenous or orally administered vitamin C (44). Such inconsistencies in findings may be attributed to the choice of study population. In that meta-analysis (44), 13 out of 18 trials enrolled patients undergoing cardiac surgery and the length of ICU stay was relatively short compared with that of the present study, suggesting a lower disease severity among patients in that study than that in the current investigation. In fact, we only included a population in whom the mortality risk was more than 5% in the control group. In support of this suspicion, a previous meta-analysis on cardiac surgical patients revealed an association of vitamin C use with a lower incidence of atrial fibrillation as well as shorter ventilation time and ICU/hospital LOS without a positive impact on in-hospital mortality (15). Therefore, such findings may underscore a potential variation in the beneficial effects of vitamin C in different populations.

A meta-analysis of RCTs and that focusing on retrospective studies can sometimes produce conflicting outcomes. For instance, a meta-analysis that included 27 RCTs and 21 observational studies reported no significant impact of vitamin C on in-hospital mortality based on the meta-analysis of the RCT subgroup (45). However, the use of vitamin C was found to be associated with a lower risk of in-hospital mortality compared to the control group in the same meta-analysis based on the subgroup of observational studies. Regarding 1-month mortality risk, although that study found a borderline positive effect on the risk of mortality with the use of vitamin C based on the meta-analysis of RCTs, there was no significant difference based on the pooled data of observational studies (45). Therefore, to better evaluate the causality by avoiding confounding factors, only RCTs were included in the current meta-analysis.

Judicious interpretation of the findings of the current meta-analysis is required because of its limitations. First, the heterogeneity from our inclusion of patients with and without sepsis/septic shock as well as our definition of critically ill individuals as those whose mortality rate was higher than 5% in the control group based on a previous study (16) may bias our findings. Nevertheless, the heterogeneity was low in our mortality rate, indicating a low risk of bias. Second, the wide variation in vitamin C dosage and frequency across our included study not only may bias our results but may also explain the lack of a universal guideline for vitamin C use in the critical care setting. Our subgroup analysis focusing on dosage suggested a favorable outcome associated with a low dosage of vitamin C, highlighting a need for further research on this issue. Third, we did not investigate the efficacy of vitamin C-based combination therapy as several meta-analyses have addressed this issue (16). Finally, despite the demonstration of a gender difference in the beneficial impact of vitamin C on disease severity (i.e., acute respiratory tract infections) (46), we are unable to analyze gender-related outcomes because of a lack of relevant data.

In conclusion, this meta-analysis focusing on critically ill adults showed a lower risk of mortality in those receiving intravenous vitamin C as a monotherapy compared to those in the placebo or standard care groups. Besides, monotherapeutic use of intravenous vitamin C was associated with shorter durations of vasopressor use and mechanical ventilation. Nevertheless, the correlation between vitamin C supplementation and a reduction in mortality rate remained inconclusive on trial sequential analysis, suggesting the need for further research to verify our findings and to identify the optimal dosage for decreasing patient mortality rate in the critical care setting.

## Data availability statement

The original contributions presented in this study are included in the article/Supplementary material, further inquiries can be directed to the corresponding author.

## Author contributions

K-CH and M-HC: conceptualization. J-YC: methodology. C-WH: software. K-CH and C-CC: validation. K-CH: formal analysis. Y-JC and C-WL: investigation. I-WC: resources. I-WC and K-CH: data curation. K-CH, I-WC, and C-KS: writing – original draft preparation and writing – review and editing. C-KS: visualization and supervision. All authors have read and agreed to the published version of the manuscript.
