# Supplementary material for: Impact of intravenous vitamin C as a monotherapy on mortality risk in critically ill patients: A meta-analysis of randomized controlled trials with trial sequential analysis
Source: Front Nutr. 2023 Mar 24;10:1094757. doi: 10.3389/fnut.2023.1094757 (PMC10083893; doi:10.3389/fnut.2023.1094757)

**Supplemental Table 1.** Search strategies for Medline

| 1 | ("critical care" or "intensive care" or "critical illness" or "critically ill" or "intensive care units" or "respiratory care units" or "systemic inflammatory response syndrome" or "sepsis" or "bacteremia" or "fungemia" or "shock" or "septic" or "cytokine release syndrome" or "respiratory distress syndrome" or "acute lung injury" or "multiple trauma" or "burn*").mp. |
| --- | --- |
| 2 | exp "Critical Illness"/ or exp "Critical Care"/ or exp "Intensive Care Units"/ or exp "Systemic Inflammatory Response Syndrome"/ or exp "Sepsis"/ or exp "Bacteremia"/ or exp "Shock"/ or exp "Respiratory Distress Syndrome"/ or exp "Acute Lung Injury"/ |
| 3 | ("ascorbic Acid" or "vitamin C").mp. |
| 4 | exp "Ascorbic Acid"/ |
| 5 | Mortality.mp. |
| 6 | exp "mortality"/ |
| 7 | (1 or 2) and (3 or 4) and (5 or 6) |
| 8 | 7 and (((randomized controlled trial or controlled clinical trial).pt. or randomi*ed.ab. or placebo.ab. or drug therapy.fs. or randomly.ab. or trial.ab. or groups.ab.) not (exp animals/ not humans.sh.)) |

**Supplemental Figure 1.** Funnel plot show a low risk of publication bias on risk of mortality.


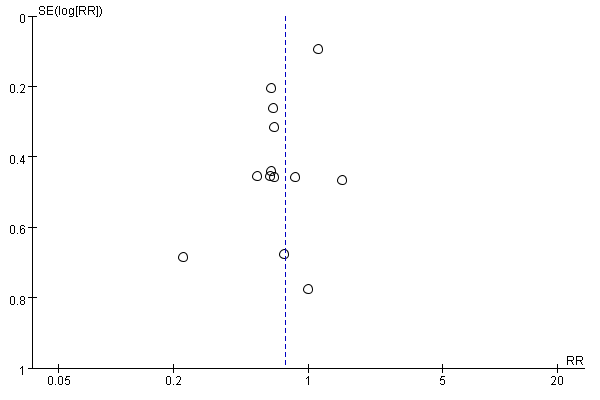


**Supplemental Figure 2.** Subgroup analysis based on treatment indication (i.e., sepsis/septic shock vs. COVID-19/pneumonia). M-H, Mantel-Haenszel; CI, confidence interval.


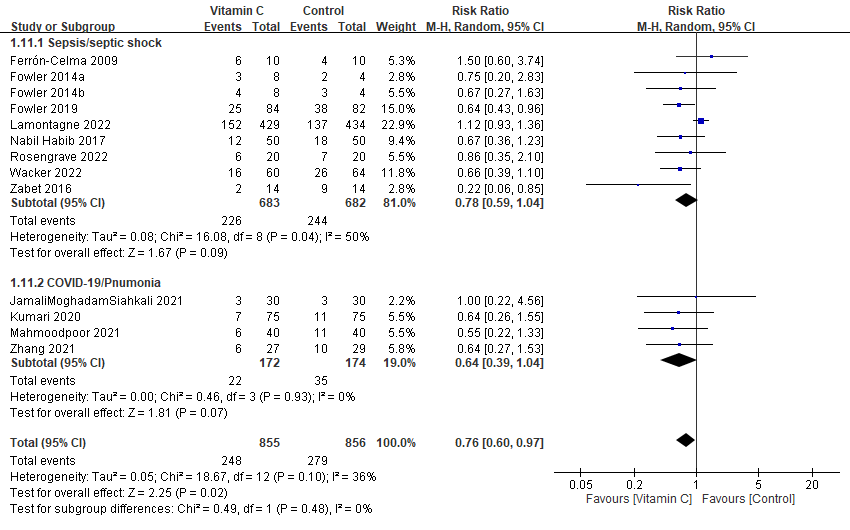


**Supplemental Figure 3.** Funnel plot showing a low risk of publication bias on length of stay in intensive care unit.


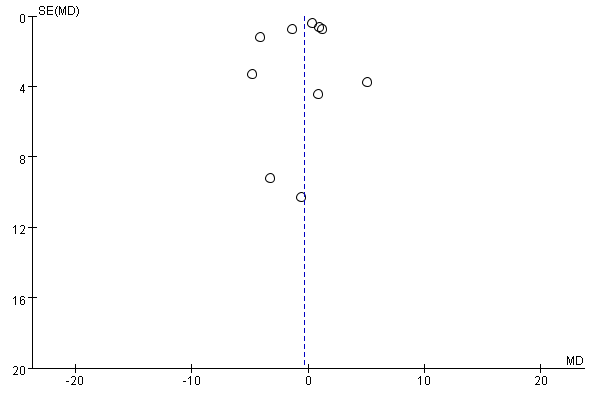

Supplement: Supplementary file 1 [file Data_Sheet_1.docx]
